# Effects of Ellagic Acid on Myocardial Contractility in Isolated and Perfused Rat Hearts

**DOI:** 10.3390/biomedicines13071645

**Published:** 2025-07-04

**Authors:** Giada Benedetti, Leonardo Carbonetti, Vincenzo Calderone, Lara Testai

**Affiliations:** 1 Department of Pharmacy, University of Pisa, Via Bonanno 6, 56126 Pisa, Italy; giada.benedetti@phd.unipi.it (G.B.); leonardo.carbonetti@farm.unipi.it (L.C.); vincenzo.calderone@unipi.it (V.C.); 2 Interdepartmental Research Center “Nutraceuticals and Food for Health”, University of Pisa, Via del Borghetto 80, 56124 Pisa, Italy

**Keywords:** ellagitannins, *Punica granatum*, myocardium, intracellular calcium stores, positive inotropism

## Abstract

**Background/Objectives**: Ellagic acid (EA) is a polyphenol found in several fruits and vegetables, including pomegranate, nuts and berries. It exhibits significant health benefits, mainly cardio- and vaso-protective; indeed, EA protects the myocardium against infarction and inhibits cardiac fibrosis. These beneficial effects may be, at least in part, promoted by calcium release from and uptake by the sarcoplasmic reticulum, which are crucial events for cardiac relaxation and contraction. Regardless, the exact mechanism is currently unclear. **Methods**: A deeper investigation of the role of EA in cardiac contractility and the underlying mechanism has been carried out by using an ex vivo model of isolated and perfused rat heart. **Results and Discussion**: EA perfusion (100 nM–10 µM) did not influence the coronary flow (CF), suggesting the absence of a vasoactivity, but significantly increased contractility parameters (LVDP and dP/dt). Interestingly, a more marked effect of EA on LVDP and dP/dt values was observed when it was perfused in the presence of AngII. Cyclopiazonic acid (CA) and red ruthenium (RR), specific antagonists of SERCA and RyRs, respectively, were used to explore the contribution of EA when the intracellular calcium handling was altered. In the presence of CA, EA, perfused at increasing concentrations, showed a very modest positive inotropism (significant only at 1 µM). Instead, RR, which significantly compromised all functional parameters, completely masked the effects of EA; furthermore, a marked reduction in CF and a dramatic impact on the positive inotropism occurred. **Conclusions**: These results demonstrate the positive inotropism of EA on isolated and perfused hearts and suggest that the RyRs may be a main target through which EA plays its effects, since inhibition with RR almost completely blocks the positive inotropism.

## 1. Introduction

Ellagic acid (EA) is a hydrolysable tannin found in free form or glycosylated in pomegranate (*Punica granatum*) fruit, but can also be found in berries and nuts [1,2,3]. After the ingestion of the fruits or juices, in the intestine, glycosylated forms are hydrolyzed by the β-glycosidases and EA can be transformed into urolithins by microbiota [4]. Although the glycosylated form and the metabolites contribute to the beneficial effects of pomegranate, EA is considered the main factor responsible for its health effects. It exhibits a series of cardiovascular effects, which primarily serve to prevent the consequences of hypertension, atherosclerosis, myocardial infarction (MI), myocardial ischemia–reperfusion (I/R), cardiac fibrosis, arrhythmia, and myopathies [5].

As concerns the possible action mechanisms underlying these cardiovascular benefits, it has been demonstrated that EA dose-dependently inhibits the ACE enzyme by about 75% at a concentration of 0.0001 mM using an in vitro approach [6,7].

These results suggest that ACE inhibition could be a central mechanism through which pomegranate juice (at least 200 mL/day) exhibits anti-hypertensive activities in humans [8,9,10,11]. However, it has been demonstrated that vasorelaxation induced by NO and by blockage of L-type calcium channels may be involved in EA cardiovascular benefits [12,13].

The contribution of NO was also analyzed via an in vivo approach, in which the administration of EA (7.5–30 mg/kg/day) reduced blood pressure, heart rate, and hindlimb vascular resistance, restored the levels of nitrate/nitrite in the blood, and improved vasorelaxation [14,15]. It should be mentioned that protection against endothelial dysfunction was also observed in small mesenteric arteries of ovariectomized hypertensive rats [16]. In agreement with these results, Benedetti and colleagues demonstrated that a *Punica granatum* extract, containing EA and punicalagins, reduced the increase in blood pressure in spontaneously hypertensive rats and protected the endothelium’s integrity [17].

In addition, EA protects the area affected by myocardial infarction and inhibits cardiac fibrosis by controlling the anti-apoptosis genes and activating the mitochondrial respiratory enzymes. Indeed, chronic treatment with EA attenuated ventricular hypertrophy by suppressing lipid peroxidation and prevented the calcium channels abnormal expression and dysfunction in pathological cardiac hypertrophy, through the reduction of oxidative stress [18]. Furthermore, EA protected against isoproterenol-induced pathological arrhythmia and suppressed the AngII-mediated hypertrophic events that can contribute to heart failure [19,20].

Some authors suggested that these beneficial events could be mediated, at least in part, through the promotion of calcium release from and uptake by the sarcoplasmic reticulum, crucial events that form the basis of cardiac relaxation and contraction [21,22]. These hypotheses may enlighten the contribution of EA on the aberrant calcium signaling typical of hypertrophic remodeling and consequent to myocardial infarction; they can also help us to understand, even if not completely, the numerous protective effects demonstrated by this tannin. Olgar and colleagues reported that EA dose-dependently reduced calcium currents with a nanomolar potency in ventricular myocytes freshly isolated from rat hearts, leading them to exhibit a negative inotropic effect that is useful in hypertension and ischemic conditions [13]. Regardless, isolated ventricular myocytes are not the best condition in which to verify the inotropism, and an in vivo or ex vivo approach would be more appropriate in order to explore the functional parameters associated with myocardial contractility and performance. In fact, the exact mechanism through which EA exerts these beneficial effects on cardiac contractility is still unclear; therefore, the aim of this study is to conduct a deeper investigation of the role of EA on cardiac contractility by using an ex vivo model of isolated and perfused hearts and examine the underlying mechanisms.

## 2. Materials and Methods

The experiments were carried out, in accordance with the European Union Council Directive 2010/63/EU, on male normotensive Wistar Kyoto rats (350–400 g). The experiments were carried out with the authorization of the Ethical Committee of the University of Pisa and of the Italian Ministry of Health (authorization number DB173.N.IXS). All the animals were housed in a room under controlled temperature (23–25 °C), humidity (50%) and lighting (12 h light/dark cycle), with food and water provided ad libitum.

**Figure 1:**
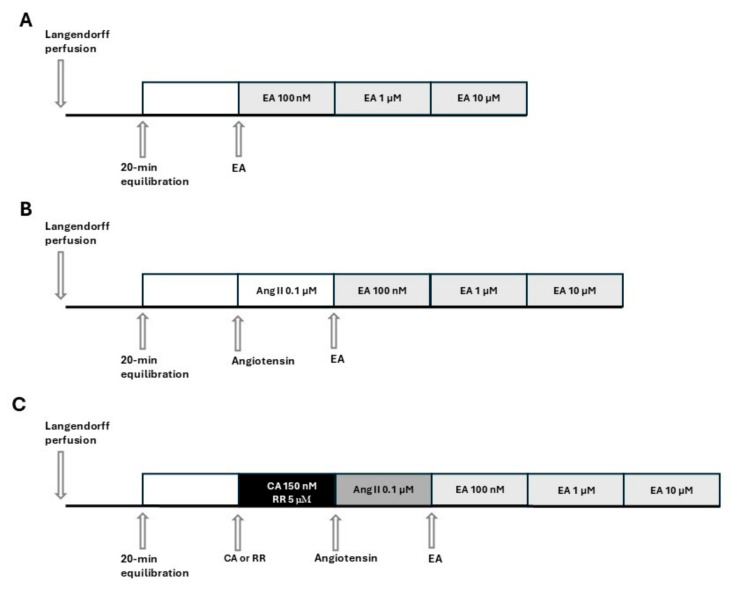
Schemes of the experimental protocols and pharmacological treatments administered to each group. (**A**): perfusion of the isolated and perfused hearts with EA at increasing concentrations. (**B**): perfusion of the isolated and perfused hearts with EA at increasing concentrations after pre-treatment with AngII (AngII was perfused for the entire experimental protocol). (**C**): perfusion of the isolated and perfused hearts with EA at increasing concentrations after pre-treatment with blockers (CA or RR) and then with AngII (blocker and AngII are perfused for the entire experimental protocol).

On the day of the experiment, the animals were euthanized with an overdose of sodium thiopental (100 mg/kg, i.p., MSD, Milan, Italy) and bled. After opening the chests, the hearts were quickly excised and placed in a 4 °C Krebs solution (composition mM: NaHCO_3_ 25.0, NaCl 118.1, KCl 4.8, MgSO_4_ 1.2, CaCl_2_·2H_2_O 1.6, KH_2_PO_4_ 1.2, glucose 11.5; Merck KGaA, Darmstadt, Germany) equilibrated with clioxicarb (a gas mixture composed of 95% plus O_2_ 5% CO_2_), to stop the contraction and reduce oxygen consumption. Rapidly, the ascending aorta was cannulated, and each heart was placed in a Langendorff apparatus and perfused under constant pressure (70–80 mmHg) with Krebs solution, maintained at 37 °C, and bubbled continuously with clioxicarb. The above procedure was completed within 2 min. In order to record functional parameters, a water-filled latex balloon, connected to a pressure transducer (Bentley Trantec, mod 800), was introduced into the left ventricle through the mitral valve and the volume was adjusted to achieve a stable left ventricular end-diastolic pressure of 5–10 mmHg. Heart rate (HR), left ventricular developed pressure (LVDP), and dP/dt (i.e., the ratio of pressure change in the ventricular cavity during the isovolemic contraction period) were continuously monitored by Biopac software 6.0 (Goleta, CA, USA). Hearts showing severe arrhythmia or instable functional values during the equilibration period were discarded. Coronary flow (CF) was volumetrically measured at intervals of 5 min. Coronary flow was expressed as ml/min, and was then normalized by the heart weight (ml/min/g). After 20 min of equilibration, the hearts were subjected to the subsequent experimental procedures. The experimental protocols and pharmacological treatments administered to each group are listed in Figure 1. Specifically, in accordance with Figure 1A, EA (Cayman Chemical Company, Ann Arbor, Michigan, USA) was perfused at increasing concentrations in the range 100 nM–10 µM. Each concentration of EA was perfused for 15 min, until a stabilization of the values was achieved. Based on Figure 1B, hearts were pre-treated by perfusion with AngII (0.1 µM, Merck KGaA, Darmstadt, Germany), and 15 min later, perfusion with EA at increasing concentrations (100 nM–10 µM) was started. Finally, based on Figure 1C, hearts were perfused for 15 min with the blocker, cyclopiazonic acid (CA), or red ruthenium (RR) (Merck KGaA, Darmstadt, Germany), subsequently pre-treated with AngII, and finally perfused with EA (100 nM–10 µM).

The baseline values of every parameter were assumed to be 100%, while the values subsequent to the pharmacological treatments were expressed as a percentage of baseline values. Then, changes in coronary flow, following the pharmacological treatments, were expressed as percentage of basal coronary flow, CF (%). LVDP was recorded as mmHg and the baseline value (recorded at the end of equilibration period) was considered as 100%. Changes in LVDP consequent to pharmacological treatment were expressed as percentage of basal LVDP. dP/dt, +dP/dt and −dP/dt, likely among the oldest measures of left ventricular performance (both contractility and relaxation), were measured in mmHg/s.

Finally, the time constant (Tau) of isovelumic relaxation is commonly used to evaluate the lusitropism of the heart and as a measurement of diastole duration. It has been expressed as a percentage of a baseline value subtracted from the vehicle value [23,24].

Each acquired value was the mean of at least 3 measurements, obtained by using 3–5 animals per group. Data were analyzed using GraphPad Prism 8.0 and expressed as mean ± SEM. Statistical analysis was carried out by one-way ANOVA and Bonferroni’s post-test. A value of *p* < 0.05 was considered as an indicator of significant difference. The symbol * indicates the significance compared to the baseline, the symbol § indicates the significance compared to AngII, whereas the symbol # indicates the significance compared to blocker (CA or RR).

## 3. Results

**Figure 2:**
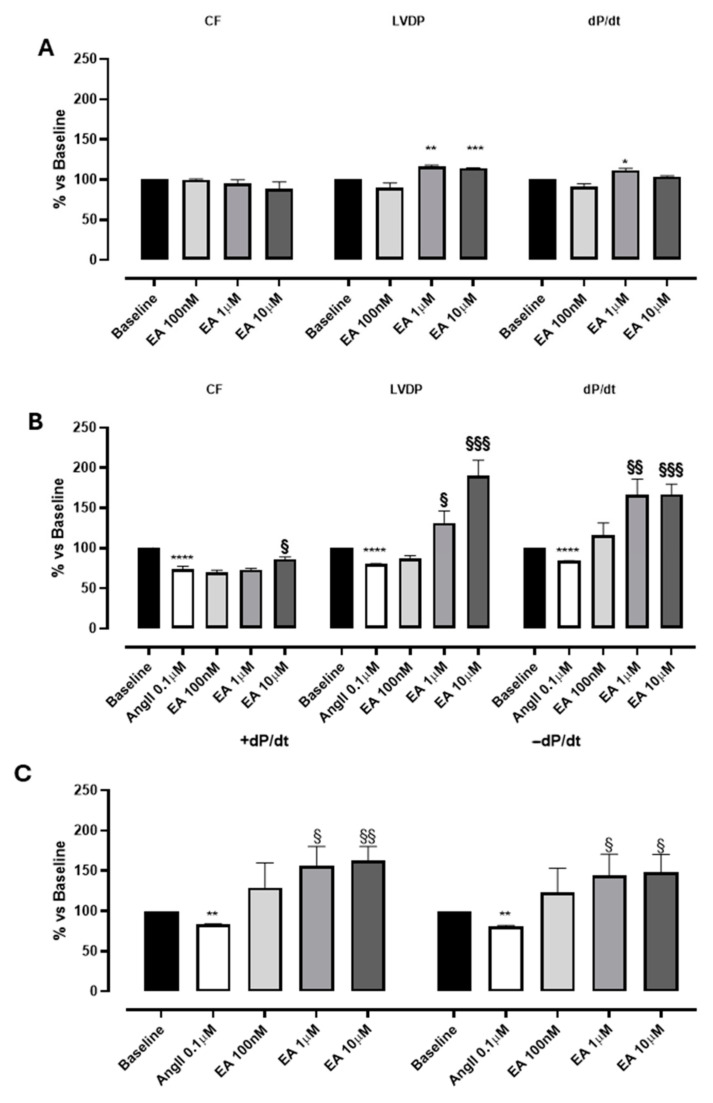
The effects of EA on coronary flow (CF), left ventricular developed pressure (LVDP) and cardiac performance (dP/dt) after perfusion at cumulatively increasing concentrations on basal (**A**) and on AngII-induced coronarospasm (**B**). (**C**) shows the effects of EA on the speed of contraction (+dP/dt) and on the speed of relaxation (−dP/dt) at cumulatively increasing concentrations. * indicates the significance compared to baseline, * *p* < 0.05, ** *p* < 0.01, *** *p* < 0.001, and **** *p* < 0.0001; § indicates the significance compared to AngII, §§ *p* < 0.001, §§§ *p* < 0.0005.

Perfusion with EA did not impact the coronary flow in the range tested (0.1–10 µM), while EA, at 1 and 10 µM, showed a significant increase in contractility parameters: LVDP and dP/dt values (Figure 2A; Table 1). Preliminary experiments carried out with the vehicle in which EA was dissolved (DMSO 1%) confirmed that it was devoid of significant effects on the evaluated parameters.

Interestingly, a more marked effect of EA was observed when it was perfused in the presence of AngII. According to the literature [25], the perfusion with AngII (0.1 μM) elicited a marked and significant reduction in CF (of about 30%), and parallelly, a variation in LVDP and the speed of contraction and relaxation (dP/dt) was reported (Figure 2B, Table 1). Indeed, AngII, when perfused on isolated hearts, can act through the activation of AT1 receptors located on the sarcolemmatic membranes of the coronary vessels, promoting a significant vasospasm, and at the same time can stimulate the AT1 receptors located on the sarcolemmatic membranes of the cardiomyocytes, exerting a positive effect on the contractility [26]. In our experimental conditions, a clear prevalence of the AngII-mediated vascular effects was observed, and the reduction in contractility can be justified by the reduction in coronary flow.

In hearts pre-treated with AngII, the perfusion with EA concentration-dependently increased both LVDP and dP/dt, more distinctly than its effects on baseline sample. Interestingly, EA achieved the maximum increase in LVDP and dP/dt at 10 µM (190 ± 19% and 167 ± 12.8%, respectively), without observable effects on CF (Figure 2A,B; Table 1). No effect on heart rate was recorded for the basal sample or AngII precontraction.

**Figure 3:**
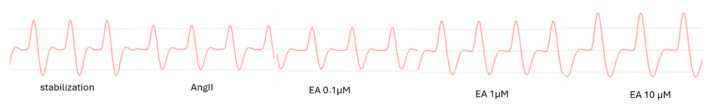
Representative picture of the effect of EA, at cumulatively increasing concentrations, on the dP/dt parameter.

Moreover, the effect of EA on the speed of contraction (+dP/dt) was almost identical to the impact on speed of relaxation (−dP/dt), suggesting that this tannin might act on both processes, increasing the performance of myocardium by about 150% (Figure 2C and Figure 3).

Finally, as regards cardiac performance, we also observed that EA, perfused after the pre-treatment with AngII, did not influence the time constant (tau), parameter which reflects the lusitropism. Thus, a positive inotropism associated with no lusitropic effect could be justified by an influence on the calcium-sensitizing system [27].

**Figure 4:**
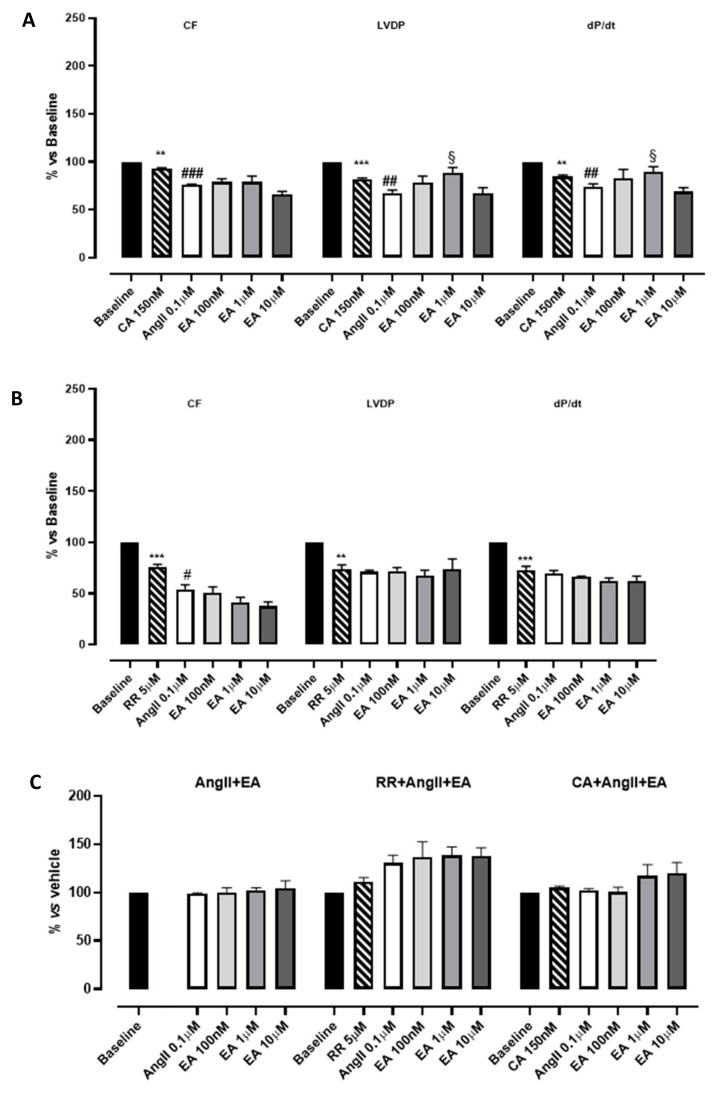
The effects of EA on coronary flow (CF), left ventricular developed pressure (LVDP) and cardiac performance (dP/dt) after perfusion at cumulatively increasing concentrations in the presence of cyclopiazonic acid (CA) (**A**) or ruthenium red (RR) (**B**). The effects of EA on the tau parameter at cumulatively increasing concentrations (**C**). § indicates the significance vs. AngII; # indicates the significance vs. the blocker (CA or RR). ^##^ *p* < 0.001, ^###^ *p* < 0.0001, ** *p* < 0.01, *** *p* < 0.001.

Hearts perfused with the inhibitor of SERCA, CA 150 nM, showed a significant reduction in functional parameters (LVDP and dP/dt) as well as CF, whereas heart rate did not appear to be modified. The subsequent perfusion with AngII caused an additive reduction in functional parameters (Figure 4A; Table 2). Then, EA, perfused at cumulatively increasing concentrations, showed a very modest positive inotropism (significant only at the concentration 1 µM). This result, together with the others reported in the literature, suggests that the impact of EA on the contractility of myocardium might be correlated with calcium handling, leading us to hypothesize that inhibition of the effective uptake of calcium into the sarcoplasmic reticulum might be deleterious for ensuring these events. As concerns the myocardial performance (dP/dt), the profile observed after perfusion with EA was exactly superimposable to that described for LVDP (Figure 4A, Table 2).

RR is described as a specific inhibitor of RyRs, which is responsible for the release of calcium from sarcoplasmic reticulum and necessary to sustain the systole. According to its role, the perfusion with RR significantly compromised the LVDP (by approximately 30%) and had a similar effect on CF and dP/dt. This reduction reached almost 50% when AngII was perfused. Increasing the concentration of EA did not improve CF; rather, it was further concentration-dependently reduced, suggesting the appearance of vascular implications. Speculating on the possible underlying pharmacological mechanisms, we suppose that the presence of RR unmasks vasoactivity. Apparently the LVDP did not change; however, a pronounced reduction in HR was caused by the treatment with the blocker; therefore, RPP value (i.e., the product between the HR and LVDP), and the CF, was reduced in a concentration-dependent manner. Under such experimental conditions, EA’s effects on cardiac performance disappeared (Figure 4B, Table 2). Preliminary experiments carried out with the vehicle in which EA was dissolved confirmed that these effects were due to the tannin.

As concerns the tau parameter, in the presence of CA, the perfusion with the tannin showed a profile almost directly equivalent to that observed in the absence of inhibitors. Instead, in the presence of RR, AngII perfusion was associated with a prolongation of the tau value and the subsequent perfusion with concentration-increasing EA further increased the tau parameter, suggesting a possible synergic effect on the lusitropic parameter (Figure 4C, Table 2).

## 4. Discussion

The results observed in this study suggest that, under our experimental conditions, EA does not exhibit a vasoactive effect, contrary to what has been reported in the literature on aortic rings and on cardiomyocytes [12], and that the improvement of cardiac contractility (both LVDP and dP/dt) might be related to a direct positive inotropic effect. It is well known that cardiac contractility is sustained by a coordinate coupling between the calcium release from and uptake by the sarcoplasmic reticulum, following the entry of calcium from sarcoplasmic L-type calcium channels. Indeed, the myocardium works as a pump and the contraction is triggered by a transient entry of calcium from the outside and a subsequent increase in the cytoplasmatic concentration of this ion through the engagement of intracellular stores. In particular, ryanodine receptors (RyRs) are responsible for the release of calcium from the sarcoplasmic reticulum and are involved in the contractility. Conversely, the sarco-endoplasmic reticulum calcium ATPase (SERCA) drives the removal of calcium from the cytosol and sustains the relaxation (diastole); in fact, defective calcium removal appears to be a key factor underlying the pathogenesis of diastolic dysfunction [28]. The free cytosolic calcium concentration following the calcium release through RyRs determines the extent of muscle activation and therefore regulates force development [29]. The sarcoplasmic reticulum plays a central role in excitation–contraction coupling and relaxation in the cardiac muscle [30]. The cytosolic calcium is then returned to the sarcoplasmic reticulum by the SERCA pump. It is responsible for the re-uptake of more than 70% of the calcium involved in the calcium transient [31], and a decrease in SERCA activity is known to be associated with various types of heart failure [32]. The balance between sarcoplasmic reticular and cytosolic calcium during both systole and diastole depends on the relative rates of these release and re-uptake processes. Altered management of calcium homeostasis at this level may be implicated in triggered arrhythmogenesis [33,34].

Traditional positive inotropic agents, including cardiac glycosides, β-adrenergic agonists, and PDE-3 inhibitors, improve cardiac contractility by increasing the calcium concentration available for contractile activation, positively affecting both chronotropism and lusitropism; on the other hand, calcium-sensitizing drugs, including levosimendan, directly augment the contractile force by increasing the affinity of troponin myofilaments for calcium, allowing a prolonged systolic interaction without significantly changing the calcium concentration or the diastolic relaxation [35,36]. Based on these premises, EA showed concentration-dependent positive inotropism, while no significant chronotropism, lusitropism, or coronary dilation were observed. Therefore, in an attempt to more deeply explore the contribution of EA in the intracellular calcium handling, we used specific antagonists of SERCA and RyRs, CA and RR, respectively.

Our results suggest that the inhibition of SERCA mitigated, but did not cancel out the effects of EA on cardiac performance (on the inotropism as well as dP/dt); while the RyRs antagonist showed a dramatic impact. The effects of EA on contractility and performance were completely deleted and vasoactivity was highlighted, contributing to a marked reduction in coronary flow.

## 5. Conclusions

In conclusion, the results obtained in this study first demonstrate the positive inotropism of EA on isolated and perfused hearts and suggest that the RyRs may be a main target through which EA exerts its effects, since the inhibition with RR almost completely blocked the positive inotropism. Future experiments will focus on a deeper comprehension of the intracellular pathways engaged, exploring the contributions of other intracellular actors, including phospholamban and troponin I phosphorylation, to evaluate EA’s contribution to calcium handling. Indeed, it cannot be excluded that EA acts via an indirect mechanism in an upstream step; in fact, this could be particularly relevant. Furthermore, drug discovery strategies will be explored to emphasize the cardioactive profile of EA and improve its pharmacokinetic profile, hopefully enhancing the enteric bioaccessibility.

## Figures and Tables

**Table 1:** The effects of cumulatively increasing concentrations of EA on coronary flow (CF), left ventricular developed pressure (LVDP), and speed of contraction (dP/dt).

Treatment	CF% vs. Baseline (Mean ± SEM)	LVDP% vs. Baseline (Mean ± SEM)	dP/dt% vs. Baseline (Mean ± SEM)
EA 100 nM	99.83 ± 1.03	89.50 ± 6.35	91.36 ± 3.55
EA 1 µM	95.12 ± 4.67	116.69 ± 1.37 *	111.67 ± 2.49 *
EA 10 µM	88.24 ± 8.88	114.44 ± 0.34	103.97 ± 0.91
AngII 0.1 µM	74.07 ± 4.51 ****	80.36 ± 0.38	84.02 ± 0.51
EA 100 nM	69.48 ± 4.00	86.59 ± 5.31	116.51 ± 14.67
EA 1 µM	73.12 ± 2.38	131.46 ± 19.03 ^§^	165.98 ± 20.09 ^§§^
EA 10 µM	86.24 ± 3.79 ^§^	190.43 ± 18.98 ^§§§§^	167.01 ± 12.78 ^§§^

* indicates the significance compared to baseline, **** *p* < 0.0001; ^§^ indicates the significance compared to AngII, ^§§^ *p* < 0.001, ^§§§§^ *p* < 0.0001.

**Table 2:** The effects of cumulatively increasing concentrations of EA on coronary flow (CF), left ventricular developed pressure (LVDP), and speed of contraction (dP/dt) in the presence of cyclopiazonic acid (CA) or ruthenium red (RR).

TREATMENT	CF% vs. Baseline (Mean ± SEM)	LVDP% vs. Baseline (Mean ± SEM)	dP/dt% vs. Baseline (Mean ± SEM)
AngII 0.1 µM	74.07 ± 4.51 ****	80.36 ± 0.38	84.02 ± 0.51
EA 100 nM	69.48 ± 4.00	86.59 ± 5.31	116.51 ± 14.67
EA 1 µM	73.12 ± 2.38	131.46 ± 19.03 ^§^	165.98 ± 20.09 ^§§^
EA 10 µM	86.24 ± 3.79 ^§^	190.43 ± 18.98 ^§§§§^	167.01 ± 12.78 ^§§^
CA 150 nM	93.11 ± 0.68	82.12 ± 1.10	84.73 ± 1.33
AngII 0.1 µM	76.27 ± 0.53 ^##^	67.55 ± 2.90	73.85 ± 3.07
EA 100 nM	78.93 ± 3.43	78.03 ± 7.12	82.57 ± 9.46
EA 1 µM	79.07 ± 6.09	88.09 ± 5.96 ^§^	89.53 ± 5.28
EA 10 µM	66.20 ± 2.98	67.19 ± 5.70	68.87 ± 4.05
RR 5 µM	77.28 ± 1.96 *	71.76 ± 3.88 **	71.27 ± 3.30 ****
AngII 0.1 µM	57.66 ± 5.08 ^#^	72.59 ± 1.96	71.02 ± 2.61
EA 100 nM	56.07 ± 6.96	74.94 ± 3.87	68.94 ± 2.83
EA 1 µM	46.51 ± 6.11	68.55 ± 4.36	63.14 ± 2.44
EA 10 µM	40.47 ± 4.49	70.48 ± 8.44	59.85 ± 4.37 ^§^

* indicates the significance vs. baseline; ^§^ indicates the significance vs. AngII; ^#^ indicates the significance vs. the blocker (CA or RR). ^##^ *p* < 0.001, ^§§^ *p* < 0.001; ^§§§§^ *p* < 0.00001, ** *p* < 0.01, **** *p* < 0.0001.

## Data Availability

Dataset available on request from the authors.
